# The Relationship of the PROMIS^®^ Pediatric Physical Activity Measure with Cardiorespiratory Fitness

**DOI:** 10.3390/children11010022

**Published:** 2023-12-24

**Authors:** Carole A. Tucker, Hannah S. Lawrence, Mary C. Hooke

**Affiliations:** 1 School of Health Professions, University of Texas Medical Branch at Galveston, Galveston, TX 77555, USA; 2 Masonic Children’s Hospital, University of Minnesota, Minneapolis, MN 55454, USA; heitk026@umn.edu; 3 School of Nursing, University of Minnesota, Minneapolis, MN 55455, USA; hook0035@umn.edu

**Keywords:** physical activity, PROMIS^®^, cardiorespiratory fitness, children, self-efficacy

## Abstract

A The PROMIS^®^ Pediatric Physical Activity (PA) measure is a new instrument with established validity that measures a child self-report on short bouts of moderate to rigorous physical activity. The purpose of this study was to explore the relationship of the PROMIS^®^ Pediatric PA item bank with cardiorespiratory fitness and self-efficacy. The study was conducted at the Minnesota State Fair. Youth ages 8 to 18 years completed the PROMIS^®^ Pediatric PA and the Self-Efficacy for PA measures on an iPad. Participants performed 3-min step test with heart rates measured 1 min posttest. Participants (N = 182) were 53% female. The PROMIS^®^ Pediatric PA had a weak, significant negative correlation with the step test measurement *(r* = −0.23, *p* = 0.001) and a weak, significant positive correlation with self-efficacy (*r* = 0.27, *p* < 0.001). Measurements did not differ between groups by sex or age group (school-age and adolescent). Youth who were obese had significantly higher heart rates post step test (*p* = 0.004); BMI percentile groups did not differ in other measures. Self-report of PA and the physiologic measure of heart rate are from two related but different physical fitness domains which supports their significant but weak relationship.

## 1. Introduction

Physical activity (PA) is part of normal daily life and occurs when there is skeletal muscle movement. Exercise is related to PA but is a sub-set of it. Exercise is planned, structured, and repetitive, such as completing repetitions of a movement to gain strength and fitness [1]. PA includes activities that are normal to childhood behaviors such as climbing on a playground, biking in a neighborhood, or running while playing tag. PA in children and adolescents advances their health and fitness. Higher levels of PA in youth are associated with improved physical fitness, cardiometabolic health, bone health, cognitive and mental health, and lower adiposity [2]. Evidence clearly supports the importance of PA for health promotion and disease prevention given the beneficial multidimensional effects of PA on cardiovascular and cardio-metabolic health; musculoskeletal health; mental health including self-concept, anxiety, and depression; and adiposity in children and youth [3,4].

PA levels are commonly assessed using wearable sensors [5,6] and/or by using self-reported measures (e.g., diaries, activity logs, or patient-reported outcome measures (PROMs)). While there are pros and cons of each method, self-reported PA approaches have proven valid, practical, and economical for large epidemiological studies assessing habitual PA [7,8,9].

The Patient-Reported Outcomes Measurement Information System (PROMIS^®^) is the product of a cooperative research initiative launched by the National Institute of Health (NIH) to develop a large set of PROMs based on a rigorous measure development process [10,11,12]. PROMIS^®^ Pediatric Physical Activity (PA) is one of the pediatric instruments developed using a mixed-methods approach [13,14,15]. The development process comprised domain conceptualization, harmonization of existing PA measures, content validation, and item bank calibration in a nationally representative sample of the pediatric population using item response theory (IRT) to enable interpretation relative to national norms [13,14,15]. The final PROMIS^®^ Pediatric PA item bank measures physical activity experiences based on constructs that are best recalled by children such as physiological response, symptoms, and short bouts of higher intensity activities. PROMIS measures generate T-scores. T-scores are standard scores with a mean of 50 and standard deviation of 10 in a reference population (usually the U.S. general population). The psychometric properties have been examined and reported to have a test–retest reliability of 0.65 and marginal reliability of 0.91 [15]. The PROMIS-PA instrument has demonstrated convergent validity with average daily steps measured by wearable devices [16]. The PROMIS^®^ Pediatric PA ten-item item bank and the eight-item short form are precise and valid instruments of self-reported physical activity in children that can be used to collect data on children’s lived experiences of short bouts of moderate to vigorous PA.

Physical activity and physical fitness are related but separate concepts. Cardiorespiratory fitness is one component of physical fitness and refers to the ability of the circulatory and respiratory systems to supply oxygen to skeletal muscles during sustained physical activity [1]. Cardiorespiratory fitness can be evaluated in adults and children with a clinical performance assessment including a “step-test”, which is an effective measurement for mass testing [17,18,19,20]. In a step-test, an individual steps up and down a step at a set pace for 1–5 min and their physiological changes (e.g., heart rate) are recorded. The timing of heart rate measurement has varied by research group but the principles of the test are consistent. In their study of 14,500 Polish school-age children, Jankowski [19] and colleagues established a reference range by measuring participants’ heart rates one minute after they had stepped up and down at a rate of 24 steps per minute.

In a review of the research, Armstrong [21] did not find a relationship between the level of PA in the everyday lives of children and VO2 max physiologic measures of aerobic fitness. In a more recent study, cardiovascular fitness in children was measured using the Progressive Aerobic Cardiovascular Endurance Run (PACER) test; researchers found that scores on the PACER were significantly associated with PA recorded in a log over a 7-day period [22]. Burden and colleagues [23] used a 20 m shuttle run test to measure cardiorespiratory fitness in young adolescents; they measured PA with an accelerometer worn over a 7-day period. Teens that had greater vigorous PA had better cardiorespiratory fitness; however, the relationship plateaus when the teens reach a maximum of 20 min of vigorous PA in a day [23]. The PROMIS^®^ Pediatric PA measures PA related to the child’s experiences with a physiological response to PA. Therefore, those who report a higher level of PA, in theory, may have higher cardiorespiratory fitness, as measured by the step test.

Self-efficacy is an individual’s belief about their ability to perform a behavior [24]. Self-efficacy is evident in individual’s lives because it influences how people behave, think, feel, and motivate themselves [25]. In their overview of the epidemiology of physical activity among US youth, Wojcicki and McAuley [26] identified self-efficacy as a consistent determinant of physical activity. The motivation process linked with self-efficacy has a role in one’s ability to participate in physical activity because when one’s beliefs about their ability to do physical activity are increased, their motivation to obtain a physically active state is increased [27]. Researchers have confirmed that in children, self-efficacy has a significant relationship with increased physical activity [27,28,29,30,31,32]. Despite the importance of self-efficacy for PA in youth, no measure currently exists to measure this concept across the range of 8–18 years, limiting our understanding of how self-efficacy and its impact on PA changes during this age range.

The purpose of this study was to assess the convergent validity of the PROMIS^®^ Pediatric PA item bank with cardiorespiratory fitness, and self-efficacy for physical activity in youth, which has not yet reported in the literature. Our primary hypothesis was that youth who reported higher levels of PA on the PROMIS^®^ Pediatric PA would have higher levels of cardiorespiratory fitness, as measured by lower heart rates on the step-test. We also examined the association between self-reported PA and self-reported self-efficacy for physical activity. Our secondary hypothesis was that youth who reported higher levels of PA on the PROMIS^®^ Pediatric PA would report higher levels of self-efficacy. Additionally, we explored differences in study measurements by age group (school-age vs. adolescent), sex, or body mass index category (underweight, healthy weight, overweight, and obese). We also explored correlations between the PROMIS^®^ Pediatric PA item bank with cardiorespiratory fitness and with self-efficacy for physical activity by age group and by sex.

## 2. Materials and Methods

### 2.1. Participants and Setting

Participants in this convenience sample were recruited for this cross-sectional study through the University of Minnesota’s Driven to Discover (D2D) research program at the Minnesota State Fair, which is held each summer [33]. This unique university-based research program provides a forum for researchers to initiate contact with and recruit fair attendees to participate in low-risk, low-burden research studies. Adult, adolescent, and child fairgoers can walk through the open front, fan-cooled research building and choose to participate in research in a fun and time-effective way. Over the course of the 12-day fair, 2 million people attend the fair, coming from both rural and urban areas. This unique research program started in 2014. Since then, nearly 300,000 fairgoers have visited the D2D building and over 103,000 enrolled in studies across a wide range of disciplines. Participants are motivated by an interest in research, pride in the state’s university, desire for the free giveaways offered to study participants, and the fun atmosphere created in the building.

Children and adolescents between the ages of 8 and 18 years who spoke English and did not have a chronic illness volunteered to participant in the study. At the study booth, eligibility criteria were posted on an easel board with the study title, “Step Up and Moove”. The easel sign listed steps the participants would need to perform to enroll. Potential child and teen participants self-identified and a study investigator verbally explained the study following an information sheet that the parent and child could take home. Participants were informed that they could stop participation at any time during the measurements. The study was approved by the University of Minnesota’s IRB.

Children and adolescents who enrolled in the study completed the study measurements at the study booth. Self-report measurements were completed on an iPad connected to the university’s Qualtrics^®^ online survey platform. Participants provided de-identified demographic data by responding to questions within the Qualtrics^®^ survey on the iPad. The participants who completed the study measurements received a University of Minnesota string backpack that contained a cow stress ball to thank them for their time.

Following a standardized protocol, members of the study team measured participants’ weight using a digital metric scale and height using a stadiometer. Body mass index (BMI) percentile was determined using the Centers for Disease Control’s BMI percentile calculator [31]. The participant’s month and year of birth were used to prevent recording the participant’s specific birthdate—a potential identifier. BMI percentiles were then categorized as follows: underweight (less than the fifth percentile), healthy weight (between the fifth percentile and the 85th percentile), overweight (between the 85th percentile and the 95th percentile), and obese (equal to or greater than the 95th percentile).

### 2.2. Measurements

#### 2.2.1. Physical Activity

The PROMIS^®^ Pediatric PA 10-item item bank, a self-report measurement, was completed by the study participants. The PROMIS^®^ Pediatric PA instrument measures self-reported physical activity in children and has been tested in a nationally representative sample of children aged 8 to 17 years [15]. The PROMIS^®^ measures and scoring manuals are publicly available online (www.healthmeasures.net, accessed on 15 June 2020). T-scores were calculated using the free PROMIS Assessment Center scoring service with a normative score of 50 to be average, 40 and below to be low, and 60 and above to be high.

#### 2.2.2. Self-Efficacy

Self-efficacy was assessed using the Physical Activity Self-Efficacy Scale (PASES). This instrument consists of eight items and measures a child’s self-report of self-efficacy related to physical activity [34]. The scale provides insight into the child’s thought process about their ability to participate in physical activity. Each item is measured on a three-point Likert-type scale with the options “No (0)”, “Not Sure” (1), and “Yes” (2). Following the instrument’s instructions, summed scores are calculated and can range from 0 to 16, with higher scores indicating higher self-efficacy. Confirmatory factor analysis demonstrated a 1-factor, 8-item version that provides statistically valid scores [34].

#### 2.2.3. Step-Test

Cardiorespiratory fitness was measured using the pediatric step test described by Jankowski and colleagues [19]. During the test, the child or teen steps up and down on an 8-inch stationery step (an aerobic step platform) at a rate of 24 steps up and down per minute for three minutes; the rate of stepping was cued by an audio metronome [19]. The test is not a “competition” to see how many steps they can take in three minutes; rather, all participants follow the same pace set by the metronome. Participants are to be stopped if their heart rate comes within 10 bpm of their age-predicted maximal heart rate (220 minus age in years), if they are unable to maintain the set stepping cadence for more than 10 s, or if they chose to stop. Heart rate is continuously monitored via a Polar wrist monitor. The participant’s heart rate is recorded during quiet standing immediately prior to engaging in the step test, is continuously monitored during the stepping test, and then recorded at one minute after the stepping test with the participant in a sitting position [19]. A lower heart rate at one minute post stepping indicates a higher level of cardiopulmonary fitness [19]. The entire step test is completed within 5 min. Conditions of the environment for testing (temperature and humidity) were recorded each hour during the study shift at the fair. Measurements were conducted on two days in August in two 5 h shifts held between 8 AM and 8 PM.

Research staff included nursing students who were trained and supervised in step-test administration by one of the researchers who was a physical therapist; competency was established by the physical therapist observing the testing. Participants observed other participants or study team members performing the test before completing it themselves.

### 2.3. Data Analysis

Descriptive statistics were used to describe the study sample and measurements (e.g., means and standard deviations for continuous variables and percentages for categorical variables). Standard procedures were used to score responses for each survey. The correlation between scores on the PROMIS^®^ Pediatric PA scale and PASES, and PROMIS and heart rates recorded one minute after the step test were assessed using Pearson product-moment correlation coefficient. To compare age sub-groups, participants were categorized by age group using designations from the American Medical Association with school-age children being 8 to 12 years and adolescents being 13 to 18 years [35]. Difference in study variables by sex and by age sub-group (school-age and adolescent) were evaluated using a *t*-test; differences in measurements by the four BMI percentile groups were compared using one-way ANOVA.

## 3. Results

### 3.1. Demographics

The demographics of the study sample can be seen in Table 1. There were a total of 182 participants with slightly more females than males. Participants enrolled in the study and completed measurements at a rate of about 90 participants per each 5 h shift. The sample was predominantly white and non-Hispanic which is consistent with the population of Minnesota [36]. The percentage of youth who were obese (10.4%) was lower than percentages cited in US data (19.3%) and the percentage of participants who were overweight (12.6%) was also lower than national rates (16.1%) [37]. There were more participants in the school-age group than the adolescent group.

### 3.2. Measurement Scores

The average scores for measurements are summarized in Table 2. Scores on the three measurements had a wide range. The PROMIS^®^ Pediatric PA scores for Minnesota children and adolescents were within the normative score range of 40 to 60. The mean heart rate of 103.39 was below both the 50th percentile boy’s mean of 113 and girls mean of 122 reported for 14,501 Polish children ages 6 to 12 years of age [19]. On the PASES summed score, potential scores can range from 0 to 16 with a higher score indicating higher self-efficacy. The mean score of 14.15 for the PASES represented 88% of a highest possible score of 100%.

Conditions in the research booth during the two sequential days of conducting the step test, were as follows: the mean temperature was 76.9° (SD 2.4) and the mean humidity was 55.1% (SD 8.7).

### 3.3. Relationships among Variables

**Figure 1:**
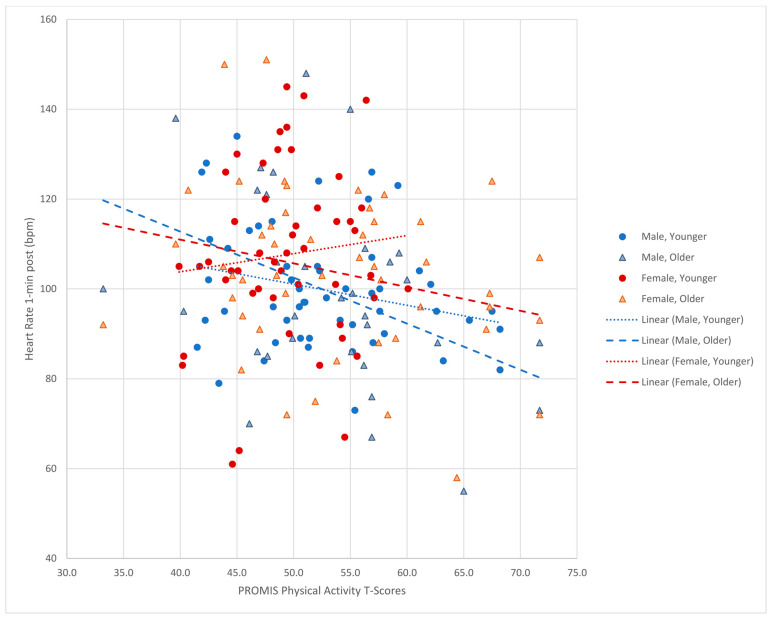
Scatterplot of PROMIS T-scores (X-axis) versus Heart Rate (beats per minute) at 1 min after steptest (Y-axis).

PROMIS^®^ Pediatric PA scores for the study sample had a weak, significant negative correlation with the heart rate one minute after completing the step test (*r* = −0.23, *n* = 182, *p* = 0.001) with higher PROMIS^®^ Pediatric PA scores associated with lower heart rates at one minute indicating a higher level of cardiorespiratory fitness (Table 3). Among the cohorts grouped by age group and sex, adolescent males had a significant negative correlation while the other cohort correlations were not significant. The school-age females’ correlation was both non-significant and in a positive direction indicating which was the opposite of the other cohorts. Scatterplots can be seen in Figure 1.

**Figure 2:**
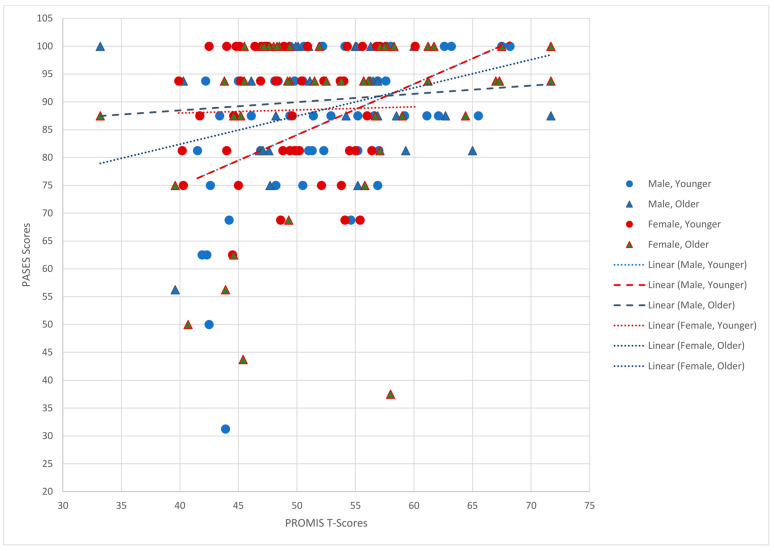
Scatterplot of PROMIS T-scores (X-axis) versus PASES Score (Y-axis).

The PROMIS^®^ Pediatric PA scores had a weak, significant positive correlation with the PASES summed score (*r* = 0.27, *n* = 182, *p* < 0.001); when PROMIS^®^ Pediatric PA T scores were higher, self-efficacy scores about physical activity were also higher. Among the cohorts grouped by age group and sex, younger males had a significant positive correlation while the other cohort correlations were not significant. A scatterplot and trend lines are provided in Figure 2.

### 3.4. Difference between Variables

The differences between sub-groups of study participants can be seen in Table 4. Males (*n* = 85) and females (*n* = 97) did not differ in any of the three study variables (PROMIS^®^ Pediatric PA, heart rate one minute after step test, and PASES). When school-age participants were compared to adolescents, there was a trend (*p* = 0.07) towards adolescents reporting higher level of activity than the younger school- age participants on the PROMIS^®^ Pediatric PA scores while other variables did not differ between the two groups. Among the four BMI percentile groups, PROMIS^®^ Pediatric PA scores and PASES summed scores were not significantly different. However, heart rates one minute after the step test were significantly higher (*p* = 0.004) in the youth who were obese compared to participants in the healthy weight group.

## 4. Discussion

A large community event, the Minnesota State Fair, was a successful location for the implementation of a population-based study of children. The recruitment and one time data collection from the convenience sample of 182 participants was accomplished in a 10-h period making it both efficient and cost effective. Participants were able to complete the study measurements without difficulty which provided a full data set for analysis.

The negative correlation between the participants’ T scores on the PROMIS^®^ Pediatric PA and their heart rates one minute after completing the step test was weak but significant. This is consistent with a study by Chen and colleagues that found a weak but significant correlation between PA self-report by youth using daily logs with performance on four physical fitness tests [22]. Correlations differed among the cohorts grouped by age group and sex with adolescent males having the significant negative correlation; this merits further investigation. The PROMIS^®^ instrument is a self-report scale that is a measure of physical activity in the past 7 days; in this case it is a measure of the respondent’s view of their own physiologic experience with physical activity. The step test is a physiologic capacity measure in which the body’s response to physical stress is evaluated. These are from two related but different domains which explains their weaker relationship. The PROMIS^®^ Pediatric PA and the PASES are also both self-report measures of related but different concepts; their positive correlation was significant but weak in strength with an *r* = 0.27; this correlation is consistent with the correlations in previous research that reported self-efficacy as significant in its relationship to a child or adolescent’s physical activity [28,29,30]. Within age group/sex cohorts, only young males demonstrated a significant positive correlation, this merits further investigation as well.

The PROMIS^®^ Pediatric PA scores did not differ significantly between boys and girls which corresponds with earlier research findings using self-report [15,32]. There was a trend for adolescents to report higher PA scores than school-age children but the difference was not significant. This finding differs from current literature that reports a decrease in PA among adolescents compared to school-age children when self-reported [15] or measured by activity sensor [15,38]. The convenience sample for this study was self-selected among youth attending the Minnesota State Fair. Potential participants observed the step test being administered as they decided if they wanted to take part in the study. Adolescents could decide to participate without a parent present as most teens attend the fair with other peers. This may have resulted in more active youth, particularly adolescents, choosing to enroll in the study. There were no significant differences in PA by weight group This is consistent with previous research by Suton and colleagues who found no differences in self-report of PA by weight groups [32]. (As the use of the PROMIS^®^ Pediatric PA measurement in large samples continues to grow, future evidence may emerge that demonstrates similarities and differences among sub-groups of children and adolescents.

Self-efficacy for physical activity did not differ between the boy and girl cohorts which differs from a previous research that found Canadian boys (*n* = 2264) reported higher levels of self-efficacy for physical activity than girls (*n* = 2623) [39]. The reason for this difference between studies could possibly be related to sociocultural (US versus Canadian) or setting (e.g., peer’s often present in our study), and may deserve additional study. Self-efficacy was not different between the adolescent and school-age groups which suggests that self-efficacy for PA may be a relatively stable construct across this age group. Among the weight groups, the PASES score was lowest in the participants who were obese but difference in these scores was not significant. This is consistent with Trost and colleague’s findings [40] that children who were obese reported lower levels of self-efficacy for physical activity.

Among the sub-groups (sex, age group, weight group) of study participants completing the 3-min step test, only the children and adolescents in the obese group had heart rates that were significantly higher indicating poorer cardiovascular health. In their study to create normative values for children completing the step test, Jankoswki and colleagues [19] also found that heart rates for overweight children were significantly higher than non-overweight children.

The study limitations must be considered when examining results. It is possible that the recruitment for our study created a selection bias favoring youth who enjoyed the challenge of a physical performance test, were more physically fit, and had less prevalence of being overweight or obese. The study measurements were continuous in the open area of the study booth and so potential participants could see others completing the step test in groups of three to four. Some groups of youth commented that it looked fun while others said that didn’t appeal to them. Additionally, in the state fair setting, the researchers had no control over the participants activities pre-study. Participants may have felt excited or anxious when taking part in the study; they could have been fatigued from walking around the 322-acre state fairgrounds; they also may eaten from the large array of food choices which could have impacted their heart rate on the step test [41].

Implementing community-based research in the state fair setting presented a unique opportunity to recruit urban, suburban, and rural youth to a low-risk study in a compressed time period. It also created some limitations. Entrance fees are expensive ($16 adult; $14 child) and additional costs for food and rides can be a limit attendance for lower income families. Attendees were also predominantly white. At the fair there are some generic “special discount” days such as Senior day and Kids day but none specific to other cultures that might occur in a different type of festival setting which would allow for recruitment of minority populations. In order to obtain IRB approval for verbal parent permission for school-age children and assent without parent permission for adolescents, the researchers deliberately avoided collecting specific identifiers related to participant’s demographics; this limited the analysis of additional variables. Although participants were screened for health criteria that would make them ineligible for the study (i.e., a chronic illness), we did not collect specific information on their health history.

## 5. Conclusions

The purpose of this study was to explore the relationship of the PROMIS^®^ Pediatric PA measures with a measure of cardiorespiratory fitness and self-reported self-efficacy for physical activity in 8–18 year olds. The PROMIS^®^ Pediatric PA demonstrated significant convergent validity with the step test; the relationship was not strong however and is reflective of the different domains used to characterize physical activity and fitness. Future research can explore how this self-report measure relates to other physiologic fitness measures as well as implement studies that recruit more diverse populations and children with chronic illness.

Children need physical activity to grow, develop, and be healthy. The PROMIS^®^ Pediatric PA measurement is a low burden instrument that can be utilized in both the research and community settings to assess physical activity in children. Assessing physical activity during health encounters can create an exercise “vital sign” which is incorporated into the child’s health record and provides a baseline for health teaching using evidence-based interventions to reduce the decline of PA reported in adolescence [42,43]. The rapid emergence and availability of digital health technologies and wearable sensors provides opportunities to combine measures of self-report and objective monitoring data to provide a more complete picture of the lived experience of PA including contexts, experiences and quantity of motion.

## Figures and Tables

**Table 1:** Participant Descriptive Characteristics (*n* = 182).

Characteristic	*n*	%
Sex		
Male	85	47
Female	97	53
Race		
White	164	90
Asian	9	5
Black-African/American & White	4	2
Black-African/American	3	2
Asian & White	2	1
Ethnicity		
Hispanic/Latino	6	3
Non-Hispanic	176	97
RUCA *		
1	140	
2	29	
3	1	
4	5	
7	1	
9	1	
10	5	
Weight status by BMI percentile		
Underweight	6	3
Healthy weight	134	74
Overweight	23	13
Obese	19	10
Age group		
School-age (8 to 12 years)	103	57
Male	53	29
Female	50	27
Adolescent (13 to 18 years)	79	43
Male	32	18
Female	47	26
	*M*	Range
Age	12.2	8–18
BMI percentile	53.4	3–99

* RUCA: USDA ERS—Rural-Urban Commuting Area Codes 1—Metropolitan area core: primary flow within an urbanized area (UA); 2—Metropolitan area high commuting: primary flow 30% or more to a UA; 3 Metropolitan area low commuting: primary flow 10% to 30% to a UA; 4 Micropolitan area core: primary flow within an Urban Cluster of 10,000 to 49,999 (large UC); 7 Small town core: primary flow within an Urban Cluster of 2500 to 9999 (small UC); 9 Small town low commuting: primary flow 10% to 30% to a small UC; 10 Rural areas: primary flow to a tract outside a UA or UC.

**Table 2:** Mean Scores, Ranges, and Standard Deviations of Measurements (*n* = 182).

Measurement	*M*	Range	*SD*
PROMIS^®^ PA ^a^ T Score	52.13	33.2–71.7	7.58
Heart rate 1 min post step test	103.39	55–222	20.25
PASES ^b^ summed score	14.15	5–16	2.10

^a^ PA = Physical activity; ^b^ PASES = Physical Activity Self-Efficacy Scale.

**Table 3:** Correlations Among Variables.

Variable	Heart Rate ^b^	PASES ^c^ Summed Score
PROMIS^®^ PA ^a^ T score All (*n* = 182)	−0.23 **	0.27 **
PROMIS^®^ PA ^a^ T score Males Age 13 to 18 years (*n* = 32)	−0.35 *	0.12
PROMIS^®^ PA ^a^ T score Female Age 13 to 18 years (*n* = 47)	−0.25	0.29
PROMIS^®^ PA ^a^ T score Males Age 8 to 12 years (*n* = 53)	−0.26	0.46 *
PROMIS^®^ PA ^a^ T score Females Age 8 to 12 years (*n* = 50)	0.10	0.02

^a^ PA = Physical activity; ^b^ = @ 1 min Post Step Test; ^c^ PASES = Physical Activity Self-Efficacy Scale; * *p* ≤ 0.05; ** *p* ≤ 0.01.

**Table 4:** Difference in Scores by Sex, Age Group, and Weight Group.

Characteristic	PROMIS^®^ PA ^a^ T Score		PASES ^b^ Summed Score		Heart Rate ^c^	
	*M*	*SD*	*M*	*SD*	*M*	*SD*
Male (*n* = 85)	52.82	7.58	14.07	2.06	100.78	21.41
Female (*n* = 97)	51.52	7.56	14.23	2.13	105.68	18.98
Difference *p* value	0.25		0.62		0.10	
Age 8 to 12 years (*n* = 103)	51.22	6.52	14.01	2.02	103.75	17.00
Age 13 to 18 years (*n* = 79)	53.30	8.66	14.34	2.19	102.92	23.93
Difference *p* value	0.07		0.29		0.79	
Under weight (*n* = 6)	52.37	4.89	14.67	2.16	110.50	17.29
Healthy weight (*n* = 134)	52.42	7.88	14.29	2.01	101.01	19.83
Overweight (*n* = 23)	51.11	8.10	14.04	2.53	102.91	20.02
Obese (*n* = 19)	51.17	5.30	13.16	1.95	118.53	18.50
Difference *p* value	0.82		0.15		<0.01	

^a^ PA = Physical activity; ^b^ PASES = Physical Activity Self-Efficacy Scale; ^c^ @ 1 min Post Step Test.

## Data Availability

The data presented in this study are available on request from the corresponding author. The data are not publicly available until completion of additional publications from the dataset.
